# A novel approach to stabilize fetal cell-free DNA fraction in maternal blood samples for extended period of time

**DOI:** 10.1371/journal.pone.0208508

**Published:** 2018-12-06

**Authors:** M. Rohan Fernando, Chao Jiang, Gary D. Krzyzanowski, Tifany Somer-Shely, Wayne L. Ryan

**Affiliations:** 1 Department of Obstetrics and Gynecology, University of Nebraska Medical Center, Omaha NE, United States of America; 2 Department of Research and Development, CFGenome Omaha NE, United States of America; 3 Methodist Physicians Clinic Women’s Center, Elkhorn, NE, United States of America; Tel Aviv University, ISRAEL

## Abstract

This study was undertaken to evaluate a novel method for stabilizing and preserving the original proportion of cell-free fetal DNA (cffDNA) in maternal blood for extended periods of time without using crosslinking agents, such as formaldehyde, which compromise DNA integrity and extraction efficiency. Blood was drawn from pregnant donors into K_3_EDTA and Blood Exo DNA ProTeck® (ProTeck) tubes. Blood drawn into both tubes were aliquoted and stored at three different temperatures. At indicated times sample aliquots were processed for cell-free DNA (cfDNA) extraction. Plasma cfDNA and cffDNA quantified by droplet digital PCR (ddPCR) assay which amplify RASSF1A gene promoter region. ProTeck reagent is formaldehyde free and inhibits blood cell metabolism in blood samples during storage. Cell-free DNA concentration increased over time in blood plasma stored in K_3_EDTA tubes at 4, 22 and 30°C. Blood stored in ProTeck tubes, cfDNA concentration was stable at 4, 22 and 30°C for 21, 28 and 7 days, respectively. In K_3_EDTA tubes cffDNA proportion decreases steadily over time whereas in ProTeck tubes cffDNA proportion remained stable. This novel technology stabilizes cffDNA proportion in maternal blood samples at 4, 22 and 30°C for 21, 28 and 7 days, respectively.

## Introduction

The presence of fetal cell-free DNA (cffDNA) in maternal blood was discovered in 1997 by Lo and colleagues [1]. After this discovery, cffDNA in maternal blood has been used as genetic material for noninvasive prenatal diagnostic and screening tests in clinical practice [2, 3, 4, 5]. Utility cffDNA for noninvasive prenatal testing is challenging because cffDNA proportion in maternal blood is very low compared to background maternal cell-free DNA (cfDNA) proportion. The median cffDNA percentage in maternal blood is 10% (range 7.8–13%) and this value further decreases with increased maternal weight due to a dilution effect caused by increased maternal background cfDNA [6]. The minimum recommended cffDNA percentage in maternal blood for accurate test results is 4%. In cases where cffDNA percentage in maternal blood is below 4%, noninvasive tests fail to provide accurate results [7, 8, 9]. Certain pre-analytical conditions such as shipping and handling of blood samples, time lapse between blood draw and sample processing and sample storage temperatures may increase maternal cfDNA background leading to significant decreases in cffDNA proportion. It has been shown that time lapse between blood draw and processing have a significant impact on cffDNA proportion in a maternal blood sample since delayed blood processing causes significant increase in maternal cfDNA background [10]. Dhallen and colleagues were the first to hypothesize that this maternal cfDNA background increase during sample handling, processing, shipping and storage was due to maternal nucleated blood cell lysis and tried to address that issue by formaldehyde mediated stabilization of nucleated blood cell membranes [11]. Another study has shown that formaldehyde can preserve the original proportion of cffDNA in maternal blood up to 36 hours at room temperature [12].

Even though formaldehyde and formaldehyde releasers are useful to stabilize blood samples they may cause other problems. Formaldehyde is known to cause protein–protein and protein–DNA crosslinks and chemically modify DNA giving sequence artifacts [13, 14, 15]. Protein–protein and protein–DNA crosslinking may reduce the efficiency of DNA extraction from plasma requiring additional incubation time with Proteinase K [16]. Previous study has reported that plasma DNA extraction from blood drawn into a commercially available blood stabilization tube requires additional incubation time with proteinase K. According to the authors of that study they modified the manufacture’s recommended protocol by increasing incubation time with Proteinase K from 30 min to 60 min at 60°C in order to reverse the effect of chemical fixation [17].

This study was undertaken to evaluate a new blood collection device which replaces crosslinking agents with metabolic inhibitors to stabilize cffDNA in maternal blood samples. It is demonstrated that with this crosslinking agent free reagent, maternal blood samples could be preserved for a longer period of time compared to claims of other commercially available blood stabilization devices.

## Materials and methods

### Pregnant donor blood samples

Blood from first trimester (8–9 weeks; LMP dating) pregnant donors were obtained from Methodist Women’s Hospital, Omaha, NE, USA. Written informed consent was obtained from all donors prior to blood draw and this study was approved (IRB # 1326) by the institutional review board of Methodist Women’s Hospital, Omaha NE. Blood was collected from each pregnant donor using standard venipuncture technique into one 10 mL K_3_EDTA tube (BD vacutainer®, Becton Dickinson, Franklin Lakes, NJ) and one 10 mL Blood Exo DNA ProTeck® tube (Catalog numbers 0019273 and 0019274, CFGenome LLC, Denver, CO, USA). Within two hours of blood collection, blood from each tube was aliquoted and stored at either 4, 22 or 30°C for different time points.

### Plasma separation

Plasma was separated from blood by two centrifugation steps as previously described by Chiu and colleagues [18]. Blood was centrifuged at 22°C at 1600 x g for 10 minutes. Then plasma layer was carefully moved without disturbing the buffy coat, to a new tube and was centrifuged at 22°C at 16000 x g for 10 minutes to remove residual cells, cell debris, apoptotic bodies and nuclei.

### Plasma DNA extraction

Manufacturer’s recommended protocol was followed to extract DNA from plasma using QIAamp® Circulating Nucleic Acid Kit (QIAGEN, Santa Clarita, CA). DNA eluted in 100 μL of elution buffer was stored at -80°C until use.

### Pregnant donor cfDNA analysis by Agilent Bioanalyzer

Blood from each tube was aliquoted into five equal aliquots and stored at 22°C and aliquots were processed at days 0, 3, 7, 14 and 28, cfDNA extracted and concentrated using a SpeedVac Concentrator (SAVANT DNA 120, Thermo Scientific, USA). Concentrated maternal cfDNA was analyzed by Agilent Bioanalyzer 2100 instrument and Agilent DNA High Sensitivity Kit following manufacturer’s recommended protocol. The Agilent 2100 Expert software analyze DNA profile of each sample automatically and displays electropherogram for each sample.

### Droplet digital PCR (ddPCR)

Plasma cfDNA and cffDNA quantification was done by a ddPCR assay designed to amplify a short segment (140 bp) of human RASSF1A gene promoter region. Forward primer 5′- AGT GCG CGC GTG AGT AGT -3′ and reverse primer 5′- GGC GAA AGT AAC GGA CCT AGT-3′ were designed using Roche ProbeFinder online software. Probe for this assay is Roche’s universal probe library probe number 24 (cat. no. 04686985001) which was recommended by the ProbeFinder. Primers were purchased from Integrated DNA Technologies (IDT) (Coralville, IA). Universal probe number 24 was purchased from Roche. A PCR master mix, 2× ddPCR™ Supermix for Probes, was purchased from Bio-Rad Laboratories (Hercules, CA). Final concentrations of primers and probe in PCR reactions were 900 nM and 250 nM, respectively, in a final volume of 20 μL. The DNA template input volume was 5 μL. A Bio-Rad QX200 Droplet Digital™ PCR System was used as described by Hindson and colleagues [19]. Thermal cycling was performed with a Bio-Rad C1000 Touch Thermal cycler. The following PCR conditions were used: 95°C for 10 min, 40 cycles of 30 s at 94°C and 50 s at 60°C followed by a heating step at 98°C for 10 min to inactivate the polymerase. Data analysis was performed using Bio-Rad QuantaSoft software version 1.7.4.0917.

### Quantification of cffDNA in maternal plasma

The promotor region of the RASSF1A gene is hypermethylated in fetal DNA and hypomethylated in maternal DNA. This difference in methylation can be used to differentiate fetal DNA from maternal DNA. Methylation-sensitive restriction enzymes cannot digest methylated regions of the genome. Therefore treating cfDNA extracted from pregnant donor blood plasma with methylation-sensitive restriction enzyme BstUI, would digest the hypomethylated promotor region of maternal RASSF1A gene leaving hypermethylated promotor region of fetal RASSF1A gene intact [20]. These undigested promotor regions of fetal RASSF1A gene can be quantified PCR amplification. Cell-free DNA extracted from pregnant donor blood plasma was treated with BstUI as previously described [20] and amplified by ddPCR that amplify RASSF1A promoter region DNA as described above.

### Data analysis

Data analysis was carried out using GraphPad Quick Calcs t test calculator online software (http://www.graphpad.com/quickcalcs/ttest1.cfm). Analysis was performed using paired, two-tailed Student's t-test and p < 0.05 was considered statistically significant.

## Results

### Inhibition of blood cell metabolism by ProTeck reagent

Glucose concentration in blood was used as an indicator of blood cell metabolism. If metabolism is inhibited by ProTeck reagent, glucose utilization is also inhibited. Blood collected into K_3_EDTA tubes showed a sharp decline in blood glucose concentration within 3 days and at day 3 glucose was undetectable (S1 Fig; supporting information section). However blood collected into ProTeck tubes showed only ~ 5% drop in blood glucose concentration within 3 days and glucose concentration was not changed from day 3 up to day 28 at 22°C (S1 Fig) indicating inhibition of blood cell metabolism by ProTeck reagent.

### Demonstration that ProTeck reagent is formaldehyde free using C^13^ NMR

First generation stabilization tubes are dependent on formaldehyde released from formaldehyde releasers. Therefore the widespread perception is that formaldehyde is indispensable for stabilization technology. We wanted to demonstrate experimentally that the ProTeck reagent is formaldehyde free and can still stabilize blood samples.) A 0.04% Formaldehyde solution (in D_2_O) was serially diluted to get concentrations of 0.02%, 0.01% and 0.005% and formaldehyde detected using C^13^ NMR. Formaldehyde gave a peak at ~ 82.58 ppm and a tiny but detectable peak was observed for 0.005% formaldehyde. Data generated were used to construct a standard curve to quantify formaldehyde in solutions. R^2^ value for standard curve was 0.9968. S2A Fig, the NMR profile obtained for 0.005% formaldehyde shows a tiny peak at ~ 82.58 ppm. Analysis of 33.3-fold diluted ProTeck reagent using C^13^ NMR showed no detectable peak at ~ 82.58 ppm indicating no or below 0.005% formaldehyde in ProTeck reagent (S2B Fig).

### Short term stability of plasma cffDNA in maternal blood at 22°C

**Fig 1:**
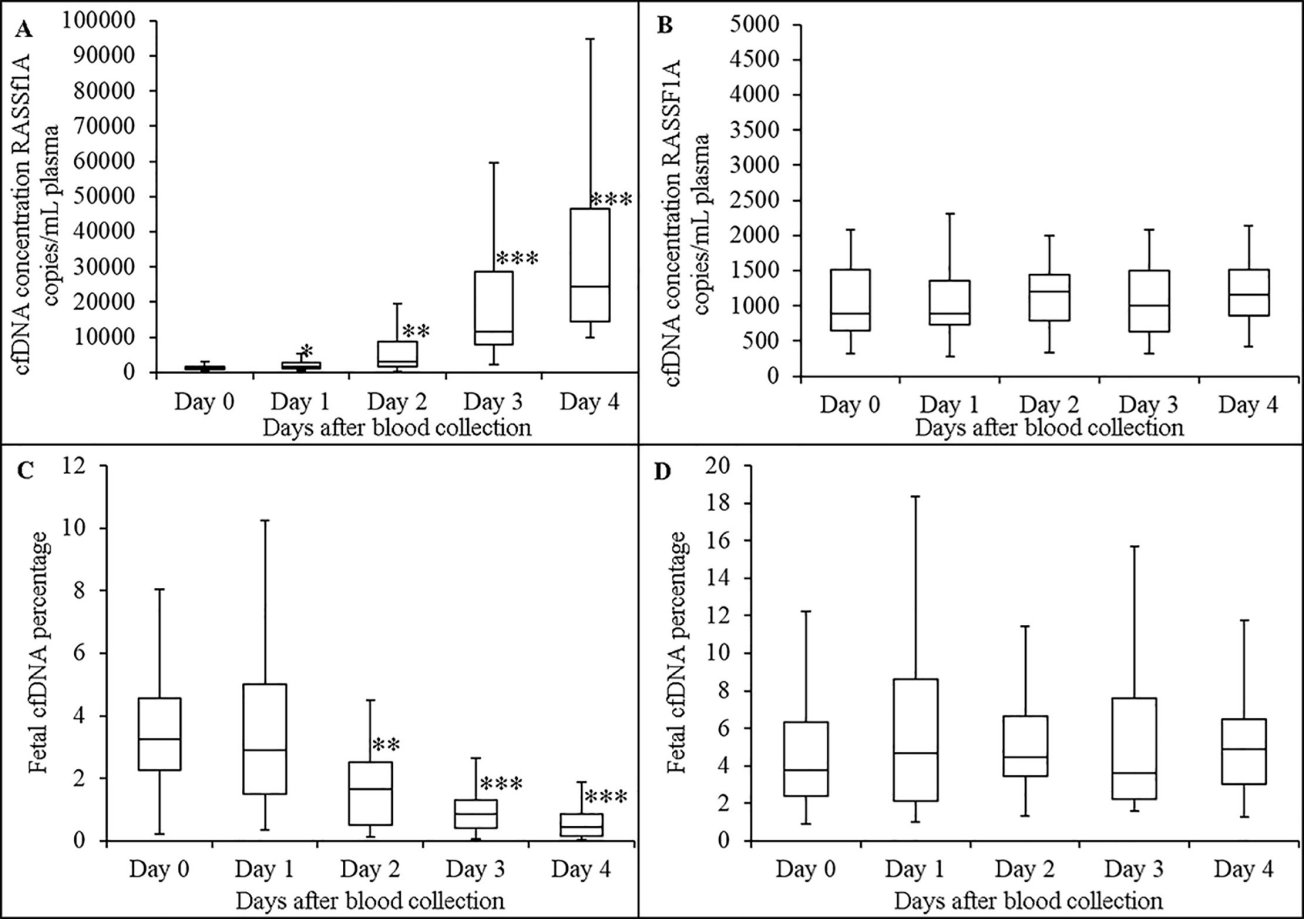
Effect of short term storage of maternal blood (at 22°C) on stability of plasma cfDNA and cffDNA proportion. Boxplot analysis of plasma cfDNA concentrations in blood drawn into K_3_EDTA (A) and ProTeck (B) tubes measured using a ddPCR assay that amplify promoter region of human RASSF1A gene. Boxplot analysis of cffDNA proportion in blood drawn into K_3_EDTA (C) and ProTeck (D) tubes. Fetal fraction was determined using ddPCR assay that amplify RASSF1A promoter region gene as described in “Material and Methods” section. The line inside of the box indicates median value. The limits of the box represent the 75th and 25th percentiles. The whiskers indicate the 10th and 90th percentiles. n = 30. cfDNA concentration for each donor is the average of two independent replicates * *p* ≤ 0.05; ** *p* ≤ 0.004; *** *p* ≤ 0.0001.

Blood from 30 pregnant donors were used for this study. Plasma cfDNA was quantified using droplet digital PCR assay that amplify a fragment of promotor region of human RASSF1A gene. Plasma median cfDNA concentrations in K_3_EDTA blood at days 0, 1, 2, 3 and 4 were 1240, 1600, 3160, 11494 and 24240 RASSF1A copies/mL plasma, respectively (Fig 1A). According to Fig 1A, there was a statistically significant increase in plasma cfDNA concentration compared to day 0 in blood stored in K_3_EDTA tubes after day 1. Plasma median cfDNA concentrations in ProTeck blood at days 0, 1, 2, 3 and 4 were 884, 884, 1200, 996 and 1156 RASSF1A copies/mL plasma, respectively (Fig 1B). According to Fig 1B, there was no statistically significant increase or decrease in plasma cfDNA concentration in maternal blood stored in ProTeck tubes. Fig 1C shows the effect of blood storage in K_3_EDTA tubes on the percentage of cffDNA in maternal blood. Plasma median cffDNA percentages at days 0, 1, 2, 3 and 4 were 3.25%, 2.9%, 1.65%, 0.85% and 0.44%, respectively. According to Fig 1C, there was a statistically significant 48.5%, 65.7% and 80% drop in cffDNA proportion in K_3_EDTA tube at days 2, 3 and 4, respectively. Fig 1D shows the effect of blood storage in ProTeck tubes on the percentage of cffDNA in maternal blood. Plasma median cffDNA percentages at days 0, 1, 2, 3 and 4 were 3.8%, 4.7%, 4.5%, 3.6% and 4.9%, respectively. According to Fig 1D, there was no statistically significant increase or decrease in plasma cffDNA percentage compared to day 0 in blood stored in ProTeck tubes.

### Long term stability of plasma cffDNA in maternal blood at 22°C

**Fig 2:**
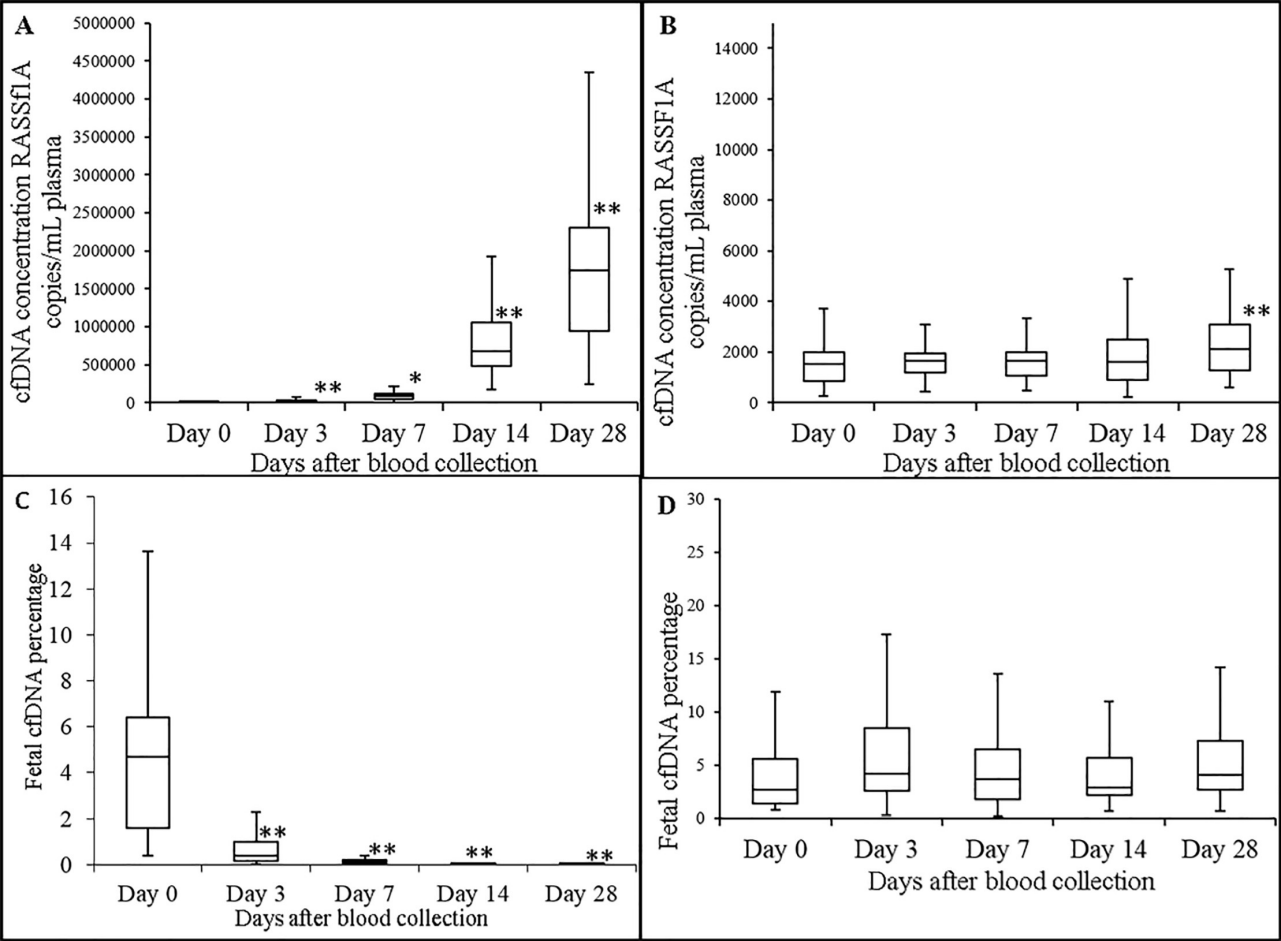
Effect of long term storage of maternal blood (at 22°C) on stability of plasma cfDNA and cffDNA proportion. Boxplot analysis of plasma cfDNA concentrations in blood drawn into K_3_EDTA (A) and ProTeck (B) tubes measured using a ddPCR assay that amplify promoter region of human RASSF1A gene. Boxplot analysis of cffDNA proportion in blood drawn into K_3_EDTA (C) and ProTeck (D) tubes. Fetal fraction was determined using ddPCR assay that amplify RASSF1A promoter region gene as described in “Material and Methods” section. The line inside of the box indicates median value. The limits of the box represent the 75th and 25th percentiles. The whiskers indicate the 10th and 90th percentiles. n = 28. cfDNA concentration for each donor is the average of two independent replicates * *p* ≤ 0.001; ** *p* ≤ 0.0004.

Blood from 28 pregnant donors were used for this study. Plasma median cfDNA concentrations in K_3_EDTA blood at days 0, 3, 7, 14 and 28 were 1560, 16520, 83480, 678800 and 1738400 RASSF1A copies/mL plasma, respectively (Fig 2A). This shows that long term storage of blood in K_3_EDTA tubes causes statistically significant increases in plasma cfDNA concentrations. Plasma median cfDNA concentration in maternal blood stored in ProTeck tubes at days 0, 3, 7, 14 and 28 were 1593, 1784, 1798, 1863 and 2331 RASSF1A copies/mL plasma, respectively (Fig 2B). According to Fig 2B, there was no statistically significant increase or decrease in plasma cfDNA concentration in maternal blood stored in ProTeck tubes except for day 28 where there was a 1.4-fold slight increase which was statistically significant (*p* = 0.0004). Fig 2C shows the effect of long term blood storage in K_3_EDTA tubes on the percentage of cffDNA in maternal blood. Plasma median cffDNA percentages at days 0, 3, 7, 14 and 28 were 4.7%, 0.4%, 0.1%, 0.008% and 0.003%, respectively. According to Fig 2C, there was a statistically significant 76%, 96%, 96% and 99.8% drop in cffDNA proportion in K_3_EDTA tube at days 3, 7, 14 and 28, respectively Fig 2D shows the effect of long term blood storage in ProTeck tubes on the percentage of cffDNA in maternal blood. Plasma median cffDNA percentages at days 0, 3, 7, 14 and 28 were 2.6%, 4.2%, 3.7%, 2.9% and 4.1%, respectively. According to Fig 2D, there was no statistically significant increase or decrease in plasma cffDNA percentage compared to day 0 in blood stored in ProTeck tubes.

### Analysis of effect of storage (22°C) on cfDNA in blood using Agilent Bioanalyzer

**Fig 3:**
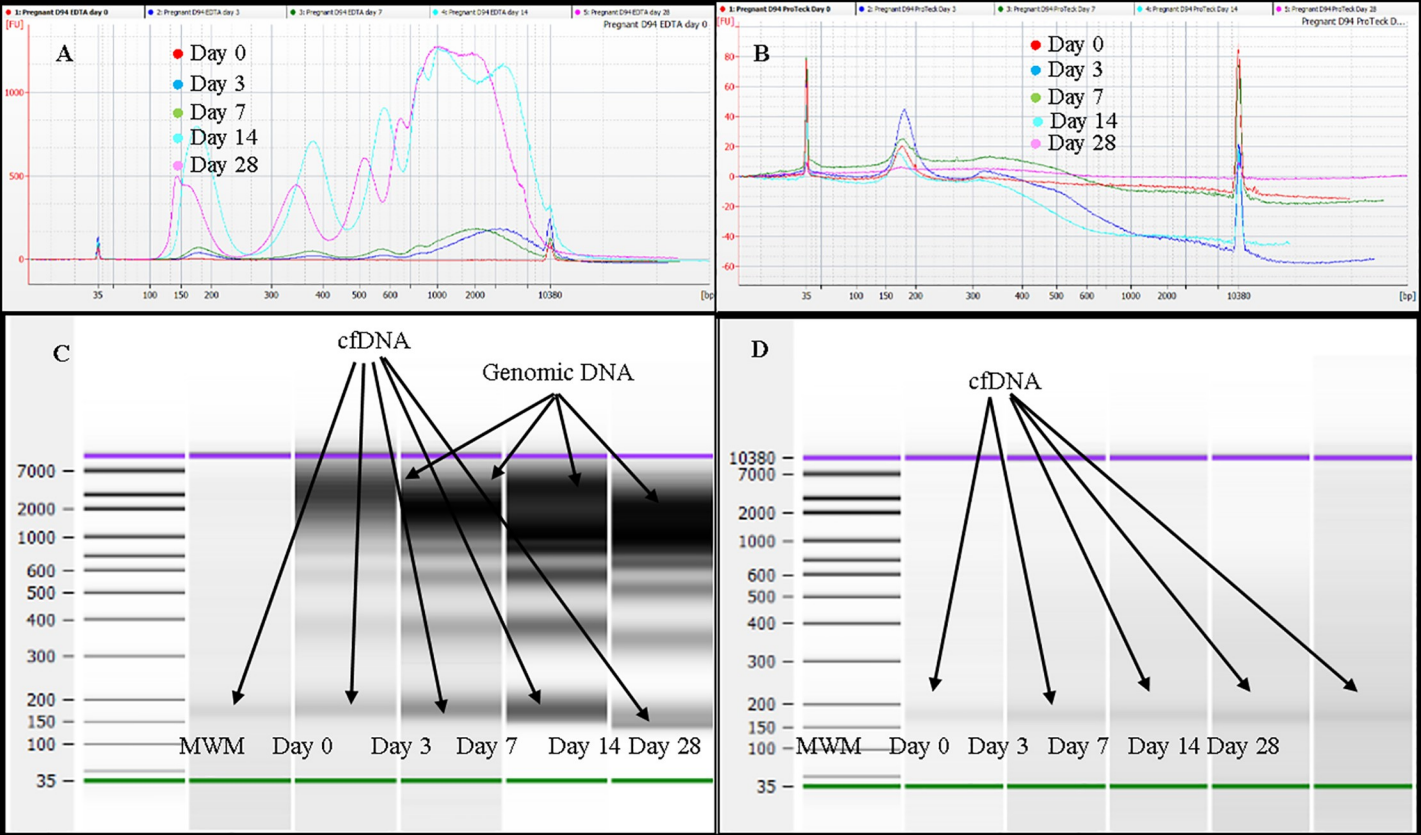
Analysis of plasma cfDNA obtained from one representative pregnant donor blood stored at 22°C using Agilent Bioanalyzer 2100 instrument and Agilent DNA high sensitivity Kit. A, Overlaid electropherograms of plasma cfDNA extracted from blood stored (at 22°C) in K_3_EDTA tubes at days 0, 3, 7, 14 and 28. B, Overlaid electropherograms of plasma cfDNA extracted from blood stored (at 22°C) in ProTeck tubes at days 0, 3, 7, 14 and 28. C, Bioanalyzer gel image for blood stored in K_3_EDTA tubes. D, Bioanalyzer gel image for blood stored in ProTeck tubes. Cell-free DNA obtained from 3 pregnant donors were analyzed. This figure shows only the results of one representative pregnant donor.

The effect of blood storage at 22°C in K_3_EDTA and ProTeck tubes on plasma DNA was studied using Agilent Bioanalyzer (Fig 3). Fig 3A shows overlaid electropherograms of plasma cfDNA extracted from blood stored in K_3_EDTA tubes at days 0, 3, 7, 14 and 28. At day 0, there was only one DNA peak between 150–200 bp ranges. No high molecular weight DNA was seen at day 0. However, days 3, 7, 14 and 28 showed additional high molecular weight peaks in addition to cfDNA peak which appears between 150–200 bp ranges. Compared to day 0 cfDNA peak height, these high molecular weight peak heights were substantially high indicating very high concentrations of contaminating high molecular weight DNA in stored plasma. Fig 3C shows Bioanalyzer gel image of K_3_EDTA plasma DNA at days 0, 3, 7, 14 and 28. According to Fig 3C, storage of blood in K_3_EDTA tube causes release of large amounts of high molecular weight DNA into plasma contaminating plasma cfDNA. Fig 3B shows overlaid electropherograms of plasma cfDNA extracted from blood stored in ProTeck tubes at days 0, 3, 7, 14 and 28. According to Fig 3B & 3D, DNA extracted from blood stored in ProTeck tubes had only one peak between 150–200 bp ranges. Unlike K_3_EDTA blood there was no high molecular weight DNA in ProTeck blood at days 3, 7, 14 and 28 indicating successful stabilization of cfDNA in blood drawn into ProTeck tubes at 22°C for 28 days.

### Stability of plasma cffDNA in maternal blood at 4°C

**Fig 4:**
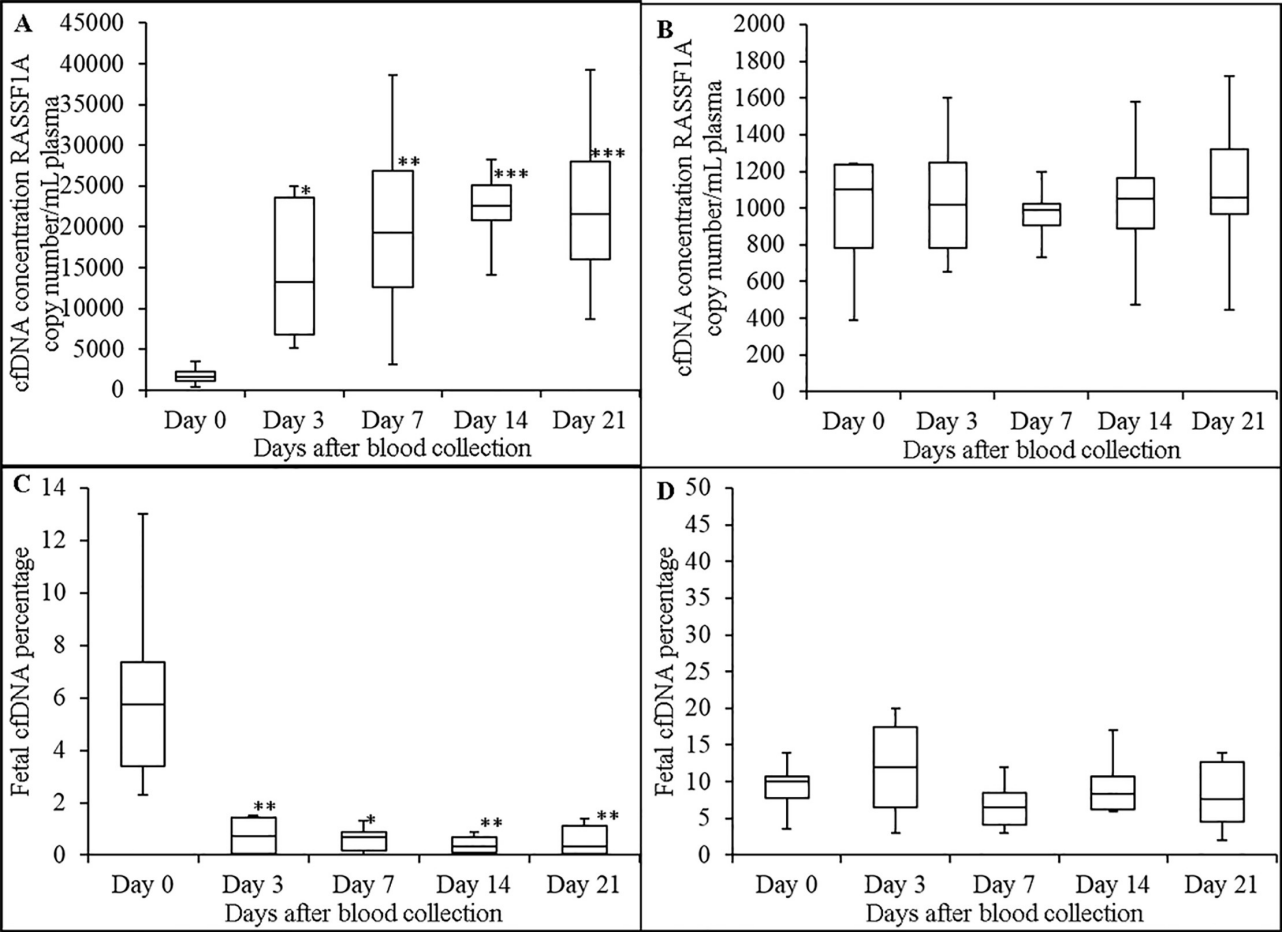
Effect of storage of maternal blood (at 4°C) on stability of plasma cfDNA and cffDNA proportion. Boxplot analysis of plasma cfDNA concentrations in blood drawn into K_3_EDTA (A) and ProTeck (B) tubes measured using a ddPCR assay that amplify promoter region of human RASSF1A gene. Boxplot analysis of cffDNA proportion in blood drawn into K_3_EDTA (C) and ProTeck (D) tubes. Fetal fraction was determined using ddPCR assay that amplify RASSF1A promoter region gene as described in “Material and Methods” section. The line inside of the box indicates median value. The limits of the box represent the 75th and 25th percentiles. The whiskers indicate the 10th and 90th percentiles. n = 7. cfDNA concentration for each donor is the average of two independent replicates * *p* ≤ 0.02; ** *p* ≤ 0.01; *** *p* ≤ 0.0008.

Blood from 7 pregnant donors were used for this study. Plasma median cfDNA concentrations in K_3_EDTA blood at days 0, 3, 7, 14 and 21 were 1580, 13260, 19360, 22630 and 21620 RASSF1A copies/mL plasma, respectively (Fig 4A). Fig 4A shows that storage of blood in K_3_EDTA tubes at 4°C causes statistically significant increases in plasma cfDNA concentrations. Plasma median cfDNA concentration in maternal blood stored in ProTeck tubes at days 0, 3, 7, 14 and 21 were 965, 1054, 1031, 1045 and 1105 RASSF1A copies/mL plasma, respectively (Fig 4B). According to Fig 4B, there was no statistically significant increase or decrease in plasma cfDNA concentration in maternal blood stored in ProTeck tubes at 4°C. Fig 4C shows the effect of blood storage in K_3_EDTA tubes on the percentage of cffDNA in maternal blood. Plasma median cffDNA percentages at days 0, 3, 7, 14 and 21 were 5.75%, 0.725%, 0.67%, 0.35% and 0.33%, respectively. According to Fig 4C, cffDNA percentage in maternal blood stored in K_3_EDTA tubes decreased significantly over time. Fig 4D shows the effect of blood storage in ProTeck tubes at 4°C on the percentage of cffDNA in maternal blood. Plasma median cffDNA percentages at days 0, 3, 7, 14 and 21 were 10%, 12%, 6.5%, 8.3% and 7.65%, respectively. According to Fig 4D, there was no statistically significant increase or decrease in plasma cffDNA percentage compared to day 0 in blood stored in ProTeck tubes at 4°C.

### Analysis of effect of storage (4°C) on cfDNA in blood using Agilent Bioanalyzer

**Fig 5:**
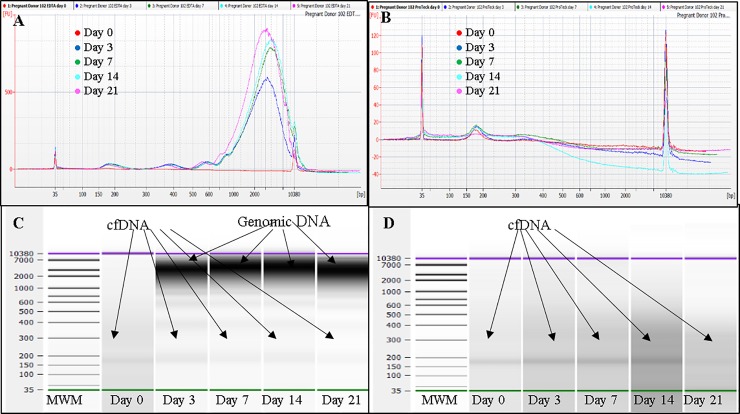
Analysis of plasma cfDNA obtained from one representative pregnant donor blood stored at 4°C using Agilent Bioanalyzer 2100 instrument and Agilent DNA high sensitivity Kit. A, Overlaid electropherograms of plasma cfDNA extracted from blood stored in K_3_EDTA tubes at days 0, 3, 7, 14 and 21. B, Overlaid electropherograms of plasma cfDNA extracted from blood stored in ProTeck tubes at days 0, 3, 7, 14 and 21. C, Bioanalyzer gel image for blood stored in K_3_EDTA tubes. D, Bioanalyzer gel image for blood stored in ProTeck tubes. Cell-free DNA obtained from 3 pregnant donors were analyzed. This figure shows only the results of one representative pregnant donor.

Fig 5A & 5C shows overlaid electropherograms and Bioanalyzer gel of plasma cfDNA extracted from blood stored in K_3_EDTA tubes, respectively. According to Fig 5A & 5C storing blood in K_3_EDTA tube does not prevent releasing high molecular weight DNA during storage. Fig 5B & 5D shows overlaid electropherograms and Bioanalyzer gel of plasma cfDNA extracted from blood stored in ProTeck tubes showing stability of cfDNA in ProTeck tubes at 4°C for 21 days.

### Stability of plasma cffDNA in maternal blood at 30°C

**Fig 6:**
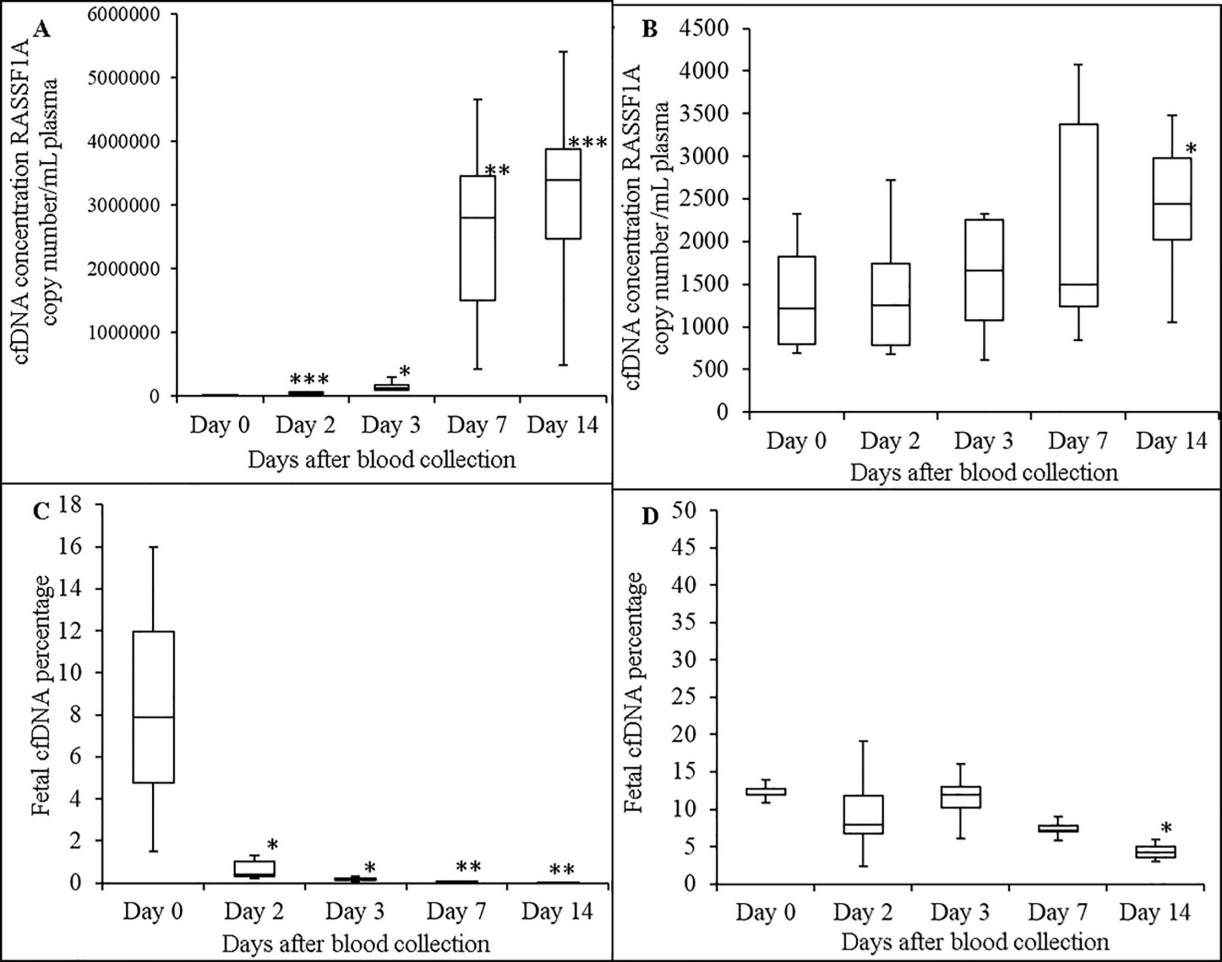
Effect of storage of maternal blood (at 30°C) on stability of plasma cfDNA and cffDNA proportion. Boxplot analysis of plasma cfDNA concentrations in blood drawn into K_3_EDTA (A) and ProTeck (B) tubes measured using a ddPCR assay that amplify promoter region of human RASSF1A gene. Boxplot analysis of cffDNA proportion in blood drawn into K_3_EDTA (C) and ProTeck (D) tubes. Fetal fraction was determined using ddPCR assay that amplify RASSF1A promoter region gene as described in “Material and Methods” section. The line inside of the box indicates median value. The limits of the box represent the 75th and 25th percentiles. The whiskers indicate the 10th and 90th percentiles. n = 7. cfDNA concentration for each donor is the average of two independent replicates * *p* ≤ 0.03; ** *p* ≤ 0.01; *** *p* ≤ 0.006.

Blood from 7 pregnant donors were used for this study. Plasma median cfDNA concentrations in K_3_EDTA blood at days 0, 2, 3, 7 and 14 were 1840, 38540, 126520, 2796000 and 3390000 RASSF1A copies/mL plasma, respectively (Fig 6A). Fig 6A shows that storage of blood in K_3_EDTA tubes at 30°C causes statistically significant increases in plasma cfDNA concentrations. Plasma median cfDNA concentration in maternal blood stored in ProTeck tubes at days 0, 2, 3, 7 and 14 were 1355, 1405, 1596, 2179 and 2742 RASSF1A copies/mL plasma, respectively (Fig 6B). According to Fig 6B, there was no statistically significant increase or decrease in plasma cfDNA concentration in maternal blood stored in ProTeck tubes at 30°C up to 7 days. However at day 14 there was a statistically significant increase compared day 0 value. Fig 6C shows the effect of blood storage in K_3_EDTA tubes on the percentage of cffDNA in maternal blood. Plasma median cffDNA percentages at days 0, 2, 3, 7 and 14 were 7.9%, 0.42%, 0.2%, 0.0065% and 0.0025%, respectively. According to Fig 6C, cffDNA percentage in maternal blood stored in K_3_EDTA tubes decreased significantly over time. Fig 6D shows the effect of blood storage in ProTeck tubes at 30°C on the percentage of cffDNA in maternal blood. Plasma median cffDNA percentages at days 0, 2, 3, 7 and 14 were 12%, 8%, 12%, 7.15% and 4.2%, respectively. According to Fig 6D, there was no statistically significant increase or decrease in plasma cffDNA percentage compared to day 0 in blood stored in ProTeck tubes at 30°C except for day 14. At day 14 there was a statistically significant decrease in cffDNA percentage.

### Analysis of effect of storage (30°C) on cfDNA in blood using Agilent Bioanalyzer

**Fig 7:**
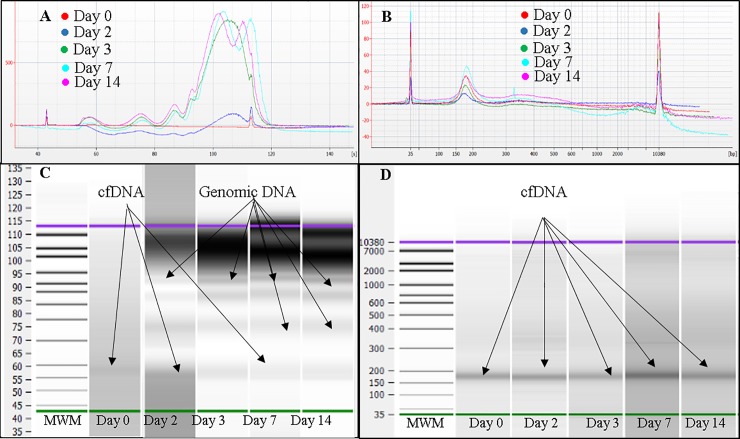
Analysis of plasma cfDNA obtained from one representative pregnant donor blood stored at 30°C using Agilent Bioanalyzer 2100 instrument and Agilent DNA high sensitivity Kit. A, Overlaid electropherograms of plasma cfDNA extracted from blood stored in K_3_EDTA tubes at days 0, 2, 3, 7 and 14. B, Overlaid electropherograms of plasma cfDNA extracted from blood stored in ProTeck tubes at days 0, 2, 3, 7 and 14. C, Bioanalyzer gel image for blood stored in K_3_EDTA tubes. D, Bioanalyzer gel image for blood stored in ProTeck tubes. Cell-free DNA obtained from 3 pregnant donors were analyzed. This figure shows only the results of one representative pregnant donor.

Fig 7A & 7C shows overlaid electropherograms and Bioanalyzer gel of plasma cfDNA extracted from blood stored in K_3_EDTA tubes, respectively. Fig 7A & 7C shows that storing blood in K_3_EDTA tubes des n not prevent release of high molecular weight genomic DNA over time. Fig 7B & 7D shows overlaid electropherograms and Bioanalyzer gel of plasma cfDNA extracted from blood stored in ProTeck tubes, respectively. According to Fig 7B & 7D, cfDNA extracted from blood stored in ProTeck tubes showed stability at 30°C for 14 days.

## Discussion

Fetal cell-free DNA present in maternal blood is being used as genetic material for non-invasive prenatal screening and diagnostic assays in clinical practice [2, 3, 4, 5]. A major limitation preventing widespread utility of cffDNA in noninvasive prenatal testing is low abundance of cffDNA in maternal blood. The minimum recommended cffDNA percentage in maternal blood for accurate test results is 4% [7, 8, 9]. Therefore it is critical that assay providers and new assay developers take appropriate measures to preserve the original proportion of cffDNA in maternal blood during the pre-analytical phase of the assay. The original proportion of cffDNA in maternal blood may decrease due to two reasons. DNase enzyme present in plasma may degrade cffDNA in maternal plasma decreasing the original proportion. The other possibility is an increase in maternal background cfDNA concentration during the pre-analytical phase of the assay which contributes towards the reduction of the original proportion cffDNA. Recent research findings show that a large proportion of cfDNA in human blood plasma is localized in membrane bound extracellular vesicles [21] which prevent degradation of plasma cfDNA by plasma DNase. Therefore the major factor that contribute towards decreasing the original proportion of cffDNA concentration in maternal blood is increased background maternal cfDNA concentration during pre-analytical phase. Angert and colleagues have experimentally shown that delayed blood processing causes significant increase in background maternal cfDNA concentration [10]. Dhallan and colleagues suggested that this increase is due to the lysis of maternal nucleated blood cells and shown that addition of formaldehyde to blood immediately after blood draw could prevent this background maternal cfDNA increase by stabilizing nucleated blood cell membranes [11]. However, recent studies suggest that cfDNA in blood increases upon blood storage mainly due to release of extracellular vesicles from viable nucleated blood cells [21].

Formaldehyde and formaldehyde releasers have been used for short term stabilization of nucleated blood cell membranes thereby preventing the increase of maternal background cfDNA concentration upon blood storage. However, formaldehyde chemically modifies DNA giving sequence artifacts [13, 14, 15] and reduces DNA extraction efficiency due to protein-protein and protein-DNA crosslinking requiring additional incubation time with Proteinase K [17]. Therefore, there is an urgent need to develop second generation technologies to stabilize the original proportion cffDNA in maternal blood without using crosslinking agents such as formaldehyde.

This study was designed to investigate a blood collection device (Blood Exo DNA ProTeck®) that contains a reagent capable of inhibiting enzymes in a blood sample [22]. Our hypothesis was that since extracellular vesicle release from cells is an active process, enzyme inhibition may provide a way to inhibit extracellular vesicle release thereby stabilizing cffDNA proportion in a maternal blood sample. Time dependent decrease of glucose in a blood sample is an indication of continuation of blood cell metabolism without any hindrance. Blood drawn in to a regular K_3_EDTA collection device shows such a time dependent glucose decrease (S1 Fig, Supporting information section). However blood drawn into ProTeck tubes showed an initial ~ 5% drop in blood glucose level and thereafter a very steady glucose concentration at 22°C for 28 days (S1 Fig). This shows that the reagent in ProTeck device inhibits metabolic activities in a blood sample for 28 days at 22°C. The initial ~5% drop indicates that it takes some time for ProTeck reagent to completely inhibit metabolic activity in the blood sample. ProTeck device is a10 mL blood collection tube which contains 300 μL of reagent. Hence, when 10 mL of blood is drawn into the tube, reagent is diluted 33.3-times. Therefore, in order to detect the presence or absence of formaldehyde in ProTeck device, we diluted ProTeck reagent 33.3-times and formaldehyde concentration determined using C^13^ NMR (S2 Fig, Supporting information section). According to S2B Fig, there was no detectable levels of formaldehyde in ProTeck reagent. Studies have been conducted to investigate the short and long term stability of plasma cfDNA concentration and cffDNA proportion in maternal blood samples. Fig 1 shows that both plasma cfDNA concentration and cffDNA proportion is stable in blood drawn into ProTeck tubes at 22°C for 4 days compared to blood drawn into K_3_EDTA tubes. Fig 2 shows long term study results which show that plasma cfDNA concentration and cffDNA proportion is stable in blood drawn into ProTeck device for 28 days at 22°C. The high standard error in boxplots may arise due to intra-assay variation (i.e., polymerase activity) and technical variability (i.e., pipetting, DNA extraction) Fig 3 shows maternal cfDNA analysis by Bioanalyzer. Electropherograms and gel images obtained from Bioanalyzer for K_3_EDTA and ProTeck blood show that blood drawn into K_3_EDTA tubes releases large quantities of high molecular weight DNA into plasma contaminating cfDNA whereas in blood drawn into ProTeck tubes there were no contaminating high molecular weight DNA in maternal plasma when incubated at 22°C for 28 days. There are several commercially available blood collection tubes for cfDNA stabilization in human blood samples. However, there are very few publications verifying the stability claims of those devices. Fernando et al reported a method to stabilize cffDNA proportion in maternal blood using a blood collection tube, Cell-Free DNA BCT^®^ for 14 days at room temperature [23]. However subsequent investigations carried out by Norton et al [17] and Wong et al [24] showed that the device is not capable of stabilizing cfDNA for 14 days at room temperature. Therefore according to publish results available at this time point ProTeck device is the only device that preserves cffDNA fraction in maternal blood for 28 days at room temperature.

Transportation of blood samples during winter time invariably expose blood samples to low temperatures. Therefore we studied the effect of low temperature storage on the stability of cffDNA proportion in maternal blood samples. Figs 4 & 5 shows that blood stored in ProTeck tubes at 4°C showed stable plasma cfDNA concentrations as well as stable cffDNA proportions up to 21 days. Diaz and colleagues have shown that when blood was stored in Cell-Free DNA BCT® devices at 4°C for 3 days there was an increase in plasma high molecular weight DNA and total cfDNA concentrations indicating the device underperforms at low temperature conditions [25]. They also reported reduced plasma volume and expanded cellular interface layer in blood stored in Cell-Free DNA BCT® tubes at 4°C (26). A study conducted by Hidestrand and colleagues have reported that shipping blood samples in Cell-Free DNA BCT® tubes at 4°C increased total cfDNA concentration leading to a significantly decreased cffDNA proportion [26].

Effect of storing blood at 30°C on cfDNA concentration and cffDNA proportion was investigated by comparing blood drawn into K_3_EDTA and ProTeck tubes. According to Figs 6 & 7 both plasma cfDNA and cffDNA proportion is stable in ProTeck tubes up to 7 days compared to K_3_EDTA tubes. However, at day 14 there was statistically significant increase in plasma cfDNA concentration leading to a statistically significant decrease in cffDNA proportion.

Molecular diagnostics is a rapidly growing field in Clinical Medicine. Next generation sequencing (NGS) and other advance technologies used in molecular diagnostics requires a lot of resources and highly skilled persons leading to increased healthcare cost. One way to reduce this higher cost is to centralize laboratories that offer molecular diagnostic assays. This centralization process demands shipping of blood samples from peripheral hospitals to large centralized laboratories. This forces cost-reducing economy of scale optimizations, such as batch-shipping and batch-processing, to occur. Further, it is critical that assay providers pay attention to the pre-analytical phase of the assay to maintain the sample integrity during the entire pre-analytical phase.

This study shows that ProTeck device can stabilize both plasma cfDNA concentration and cffDNA proportion in maternal blood samples. Fetal cfDNA proportion is stable at 22°C for up to 28 days, at 4°C for up to 21 days and at 30°C for 7 days. ProTeck reagent is free of formaldehyde which eliminates prolonged incubation times with Proteinase K and sequence artifacts caused by formaldehyde. Preservation of the cffDNA proportion for extended periods of time and at broader temperature range (4–30°C) allows increased testing accuracy that is in alignment with clinical guidelines. Maintenance of cffDNA proportion for extended periods of time limits the need for re-testing for a test that is already considered time sensitive.

## Supporting information

S1 Fig Effect of ProTeck reagent on blood glucose concentration at room temperature (22°C). Blood drawn into K_3_EDTA (▲) and ProTeck (●) tubes were stored at 22°C and glucose concentration determined at indicated times as describes in “Material and Methods” section.
(TIF)

S2 Fig Analysis of ProTeck reagent by C^13^ NMR for detection of formaldehyde. ProTeck reagent was diluted 33.3-times in D_2_O and analyzed by C^13^ NMR as described in “Material and Methods” section. A, Analysis of a 0.005% formaldehyde solution by C^13^ NMR. A peak corresponding to formaldehyde appears at ~ 82.58 ppm. B, analysis of ProTeck reagent by C^13^ NMR. No peak was observed at ~ 82.58 ppm indicating no formaldehyde in ProTeck reagent.
(TIF)

S1 File Detection of inhibition of blood cell metabolism in a blood sample upon storage. Inhibition of blood cell metabolism in a blood sample was determined by determining blood glucose concentration in a blood sample upon storage.
(DOCX)

S2 File C^13^ NMR. C^13^ NMR was employed to detect formaldehyde in ProTeck reagent.
(DOCX)
