# Supplementary figures and images for: A novel approach to stabilize fetal cell-free DNA fraction in maternal blood samples for extended period of time
Source: PLoS One. 2018 Dec 6;13(12):e0208508. doi: 10.1371/journal.pone.0208508 (PMC6283530; doi:10.1371/journal.pone.0208508)

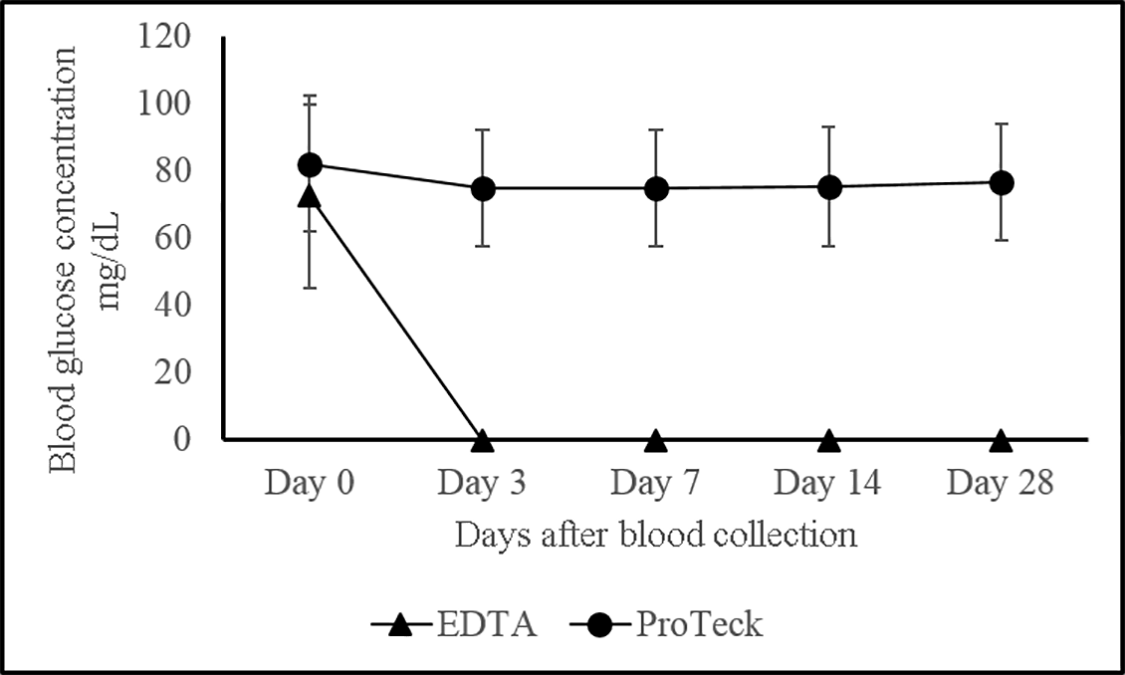

Supplement: S1 Fig — Blood drawn into K3EDTA (▲) and ProTeck (●) tubes were stored at 22°C and glucose concentration determined at indicated times as describes in “Material and Methods” section. (TIF) [file pone.0208508.s001.tif]

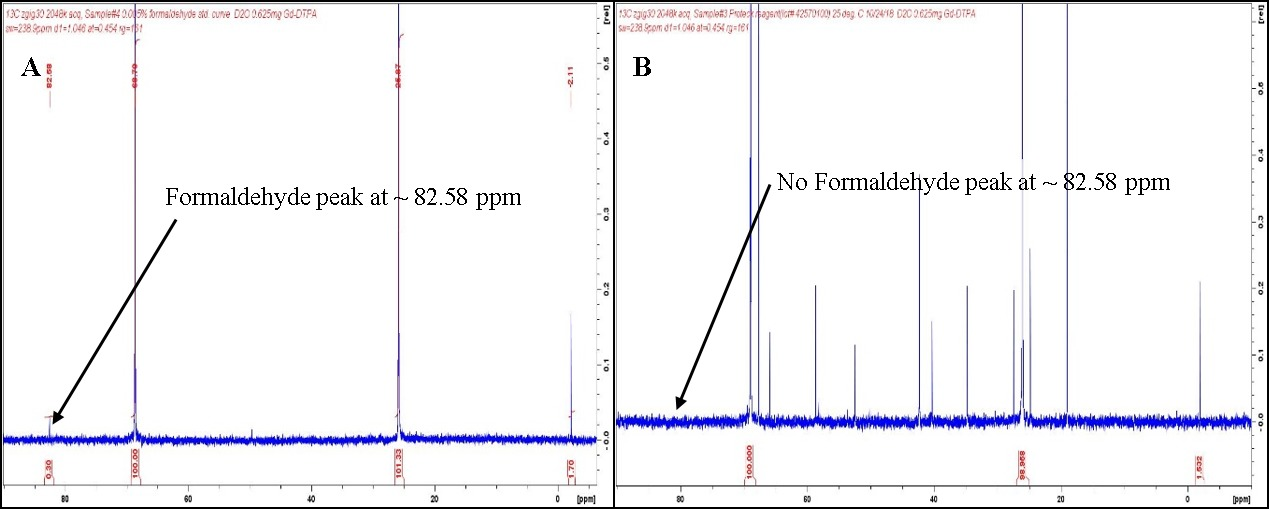

Supplement: S2 Fig — ProTeck reagent was diluted 33.3-times in D2O and analyzed by C13 NMR as described in “Material and Methods” section. A, Analysis of a 0.005% formaldehyde solution by C13 NMR. A peak corresponding to formaldehyde appears at ~ 82.58 ppm. B, analysis of ProTeck reagent by C13 NMR. No peak was observed at ~ 82.58 ppm indicating no formaldehyde in ProTeck reagent. (TIF) [file pone.0208508.s002.tif]
