# Supplementary material for: A novel approach to stabilize fetal cell-free DNA fraction in maternal blood samples for extended period of time
Source: PLoS One. 2018 Dec 6;13(12):e0208508. doi: 10.1371/journal.pone.0208508 (PMC6283530; doi:10.1371/journal.pone.0208508)
Supplement: S1 File — Inhibition of blood cell metabolism in a blood sample was determined by determining blood glucose concentration in a blood sample upon storage. (DOCX) [file pone.0208508.s003.docx]

*Blood glucose concentration*

Blood glucose concentrations were measured using Accu-Chek Aviva (S/N 45521272964) blood glucose meter and Accu-Chek Aviva plus strips (Roche diagnostics) following manufacturer’s instructions. Before glucose measurement blood samples were mixed well.
