# Supplementary material for: A novel approach to stabilize fetal cell-free DNA fraction in maternal blood samples for extended period of time
Source: PLoS One. 2018 Dec 6;13(12):e0208508. doi: 10.1371/journal.pone.0208508 (PMC6283530; doi:10.1371/journal.pone.0208508)
Supplement: S2 File — C13 NMR was employed to detect formaldehyde in ProTeck reagent. (DOCX) [file pone.0208508.s004.docx]

*C^13^ NMR analysis*

C^13^ NMR was employed to detect formaldehyde in ProTeck reagent using a previously described method (19). NMR data was acquired on a Bruker Avance III HD 600 MHz spectrometer equipped with a triple resonance HCN cryoprobe. Acquisition parameters were previously described (19). Briefly, an inverse gated carbon detector with proton decoupling sequence (zgig30) was acquired over a 36KHz sweep width, with 32,768 time domain data points ( zero-filled to 131K data points upon Fourier transform), 0.45 s acquisition time, 1 s relaxation delay, and signal averaged over 2048 acquisitions at a probe temperature of 298 Kelvin. Data were processed in Bruker Topspin 3.2.6 software with a 1 Hz exponential line-broadening factor, baseline corrected, and integrated with operator optimization of slope and bias for accurate quantization of peak volumes.
